# Socioeconomic Status, Diet, and Behavioral Factors and Cardiometabolic Diseases and Mortality

**DOI:** 10.1001/jamanetworkopen.2024.51837

**Published:** 2024-12-20

**Authors:** Peilu Wang, Xiang Gao, Walter C. Willett, Edward L. Giovannucci

**Affiliations:** 1 Department of Nutrition and Food Hygiene, School of Public Health, Institute of Nutrition, Fudan University, Shanghai, China; 2 Department of Epidemiology, Harvard T.H. Chan School of Public Health, Boston, Massachusetts; 3 Department of Nutrition, Harvard T.H. Chan School of Public Health, Boston, Massachusetts

## Abstract

**Importance:**

It remains unclear how socioeconomic status (SES) is related to the association between diet and health, as well as the role of behavioral factors, in explaining socioeconomic disparities in health outcomes.

**Objective:**

To investigate the associations of neighborhood and individual SES factors, as well as behavioral factors, particularly dietary pattern, with health outcomes.

**Design, Setting, and Participants:**

This prospective cohort study included US health professionals without chronic diseases at baseline who were enrolled in the Health Professionals Follow-Up Study (calendar years 1988-2018), the Nurses’ Health Study (calendar years 1992-2018), and the Nurses’ Health Study II (calendar years 2001-2019). Data analysis was performed in September 2023.

**Exposures:**

Repeated questionnaires were used to assess neighborhood and individual SES factors and behavioral factors, including dietary pattern (assessed using the Alternative Healthy Eating Index 2010), alcohol intake, body mass index, cigarette smoking, physical activity, sedentary television-viewing time, and sleep duration.

**Main Outcomes and Measures:**

The main outcomes were incident major cardiovascular disease (CVD), type 2 diabetes (T2D), and total mortality. The associations of SES and behavioral factors with outcomes were analyzed using multivariable Cox proportional hazards regression models with hazard ratios (HRs) per 10th- to 90th-percentile increments.

**Results:**

The study analyzed 152 192 participants for major CVD (mean [SD] age, 52.0 [8.7] years; 125 959 female [82.8%]), 151 217 participants for T2D (mean [SD] age, 52.0 [8.6] years; 125 231 female [82.8%]), and 141 145 participants for mortality (mean [SD] age, 51.6 [8.5] years; 117 627 female [83.3%]). A healthy dietary pattern was inversely associated with risk for major CVD (HR, 0.87 [95% CI, 0.82-0.93]), T2D (HR, 0.79 [95% CI, 0.75-0.84]), and total mortality (HR, 0.84 [95% CI, 0.81-0.88]). Without adjusting for neighborhood and individual SES factors, the HRs were 0.85 (95% CI, 0.80-0.91) for risk for major CVD, 0.78 (95% CI, 0.74-0.82) for T2D, and 0.82 (95% CI, 0.79-0.85) for total mortality. Neighborhood SES was inversely associated with risk for major CVD (HR, 0.90 [95% CI, 0.85-0.95]), T2D (HR, 0.92 [95% CI, 0.88-0.97]), and total mortality (HR, 0.91 [95% CI, 0.88-0.94]). Behavioral factors accounted for a large proportion of the associations with risk for major CVD (46.3% [95% CI, 32.5%-60.6%]), T2D (77.4% [95% CI, 64.5%-86.6%]), and total mortality (42.8% [95% CI, 32.9%-53.3%]).

**Conclusions and Relevance:**

In this prospective cohort study of health professionals, associations between diet and health outcomes remained similar without adjusting for SES factors, while health behaviors, including diet, accounted for a large proportion of the associations between neighborhood SES and health. These findings highlight the importance of health behaviors, particularly high-quality diets, in promoting individual health and possibly reducing health disparities associated with SES.

## Key Points

**Question:**

How does socioeconomic status (SES) relate to the association between diet and health, and to what extent do health behaviors account for the socioeconomic disparities in health outcomes?

**Findings:**

In this cohort study of 152 192 US health professionals for major cardiovascular disease, 151 217 for type 2 diabetes, and for 141 145 for total mortality, multivariable associations between dietary pattern and long-term health outcomes demonstrated minimal change without adjusting for individual and neighborhood SES. Behavioral factors, including diet, accounted for a significant proportion of the associations of neighborhood SES with health outcomes.

**Meaning:**

These findings highlight the importance of health behaviors, particularly high-quality diets, in promoting individual health and potentially reducing health disparities associated with SES.

## Teaser

This cohort study investigates the association of socioeconomic status with diet and health as well as the association of behavioral factors with socioeconomic disparities in health outcomes.

## Introduction

Observational studies have provided key evidence for associations between diet and long-term health outcomes because randomized clinical trials are often not feasible. However, these findings have been criticized for methodologic limitations due to observational designs.^1,2,3,4^ In particular, dietary patterns are associated with socioeconomic status (SES), an important health determinant. People with lower educational or income levels tend to consume excessive amounts of energy-dense and nutrient-poor foods but inadequate amounts of fresh fruits and vegetables, with potentially different patterns across neighborhoods.^5,6,7^ However, to what extent SES is involved in observed associations between diet and health outcomes remains unclear.

Additionally, the pathways through which neighborhood SES may factor into health behaviors that could contribute to health disparities are not well understood.^8^ A growing body of evidence has shown associations of neighborhood SES with health behaviors, such as diet quality, smoking, and physical activity, as well as cardiometabolic health.^6,9,10,11,12,13^ However, few longitudinal studies have investigated the extent to which behavioral factors, especially dietary patterns, might explain the association of neighborhood SES with health outcomes.

We used data from 3 US cohorts of health professionals to investigate how SES at both individual and neighborhood levels might factor into the associations between diet and health outcomes and how diet and other behavioral factors might mediate the associations between neighborhood SES scores and health outcomes.

## Methods

### Study Population

This cohort study used data from 3 ongoing prospective cohorts of health professionals. The Nurses’ Health Study (NHS) included 121 700 female registered nurses, aged 30 to 55 years, beginning in 1976.^14^ The NHS II included 116 429 female registered nurses, aged 25 to 42 years, beginning in 1989.^14^ The Health Professionals Follow-Up Study enrolled 51 529 male health professionals, aged 40 to 75 years, beginning in 1986.^15^ Participants of the 3 cohorts provided information on lifestyle and medical history through self-administered questionnaires biennially. The study was approved by the institutional review boards of the Brigham and Women’s Hospital, Harvard T.H. Chan School of Public Health, and those of participating registries as required. The institutional review boards allowed participants’ written completion of questionnaires to be considered as implied consent. We followed the Strengthening the Reporting of Observational Studies in Epidemiology (STROBE) reporting guideline.

Dietary intake during the preceding year was assessed through validated semiquantitative food-frequency questionnaires every 4 years. Participants’ residential addresses were geographically coded to derive neighborhood SES factors. Follow-up rates were approximately 90% in all 3 cohorts. In this study, we used calendar years 1992 as the baseline for the NHS (through 2018 for follow-up), 2001 for the NHS II (through 2019 for follow-up), and 1988 for the Health Professionals Follow-Up Study (through 2018 for follow-up) when SES and behavioral factors were first available.

We excluded participants who had died; those with a history of cardiovascular disease (CVD), type 2 diabetes (T2D), or cancer; those with missing data on SES factors, dietary patterns, or other behavioral factors; those with implausible energy intake; and those with an extremely high body mass index (BMI) at baseline. Participants were censored when they were 80 years old to reduce potential reverse causation (eFigure 1 in Supplement 1).

### Assessment of SES

Neighborhood SES data were extracted and updated biennially by mapping census data from the Neighborhood Change Database to participants’ residential addresses.^16^ The neighborhood SES score was computed as the sum of 9 *z* score–standardized census tract–level variables selected by principal component analysis as previously reported.^17^ Data on individual SES factors were assessed and updated through biennial questionnaires that included marital status, living arrangement, working status, parental occupations, husband’s educational level for women, and household income (eMethods in Supplement 1).

### Assessment of Diet

Participants reported consumption frequency and portion size of more than 130 items. Total energy and nutrient intake were estimated by summing nutrient content of each contributing food, multiplied by its consumption frequency. The food-frequency questionnaire–measured dietary patterns have been validated against multiple dietary records in previous studies.^18,19,20^ Diet quality was assessed using the Alternative Healthy Eating Index 2010, which consists of 11 components, scored from 0 (unhealthiest) to 10 (healthiest).^21^ To have a finer adjustment, we removed the alcohol component from the dietary pattern and adjusted for alcohol intake separately.

### Assessment of Other Factors

Information on other factors was extracted from baseline and biennially collected questionnaires that were self-administered (eMethods in Supplement 1). Race and ethnicity and family history of diabetes, cancer, or CVD were self-reported. Race and ethnicity were categorized as non-Hispanic White (hereinafter, White) and other race or ethnicity (hereinafter, racial or ethnic minority individuals [which included American Indian or Alaska Native, Asian, Black, Hispanic, and Native Hawaiian or Other Pacific Islander]) to increase statistical power because the participants were predominantly White; these data were included because race and ethnicity are often associated with social determinants of health and dietary factors. BMI was computed as weight in kilograms divided by height in meters squared. The following factors were modeled as time-varying variables: age, BMI, cigarette smoking, physical activity, sedentary television-viewing time, sleep duration, multivitamin use, regular aspirin use, regular nonsteroidal anti-inflammatory drug use, and postmenopausal hormone use for women. Missing covariates were assigned with nonmissing values from preceding questionnaires.

### Outcome Ascertainment

Primary outcomes were incident major CVD, T2D, and total mortality. Cause-specific deaths were examined as secondary outcomes. Self-reported coronary heart disease and stroke were confirmed through medical records as previously described.^22^ T2D was confirmed by supplementary questionnaires, the confirmation rate of which was estimated to exceed 97%.^23^ Deaths were identified through reports returned from next-of-kin or the postal office and through searches of the National Death Index^24^; the ascertainment rate using these methods was estimated to be 98%.^25^ The death certificate, medical records, and data from tumor registries were reviewed by clinicians who were blinded to the exposure to confirm the cause of death using the *International Classification of Diseases, Revision 8,* and the *International Classification of Diseases, Ninth Revision* (eMethods in Supplement 1).

### Statistical Analysis

Participants were followed from baseline assessment until the occurrence of the outcome, death, age 80 years, the last questionnaire response (for analysis on T2D), or the end of follow-up, whichever came first. Data analysis was performed in September 2023. We calculated the cumulative averages of dietary pattern and neighborhood SES scores. A 4-year lag was applied in the mortality analysis to reduce potential reverse causality. Both dietary pattern and neighborhood SES scores were winsorized at the 0.5th and 99.5th percentile to minimize the influence of outliers. Analyses were performed in the pooled data of 3 cohorts using Cox proportional hazards regression models, with age as the time scale. All models were stratified by age, calendar year, and cohort. Exposures were modeled as continuous variables and standardized by the 10th to 90th percentile.

We assessed how neighborhood and individual SES, as well as other risk factors, factored into the associations between dietary pattern and health outcomes by omitting 1 risk factor at a time, either individually or compositely, from the fully adjusted model while adjusting for all other variables. The relative change in HRs was quantified using the ratio of HR (RHR), as previously described,^26,27,28,29^ by comparing the multivariable adjusted model without the specific risk factors with the fully adjusted model. A large difference between the RHR and unity suggests that the omitted risk factors may substantially alter the association between dietary pattern and health outcomes. The robustness of diet and disease associations to potential uncontrolled confounding was evaluated using the E-value,^30^ which represents the minimum relative risk association needed for an unmeasured confounder with both diet and disease to account for the observed diet and disease association. We assessed how behavioral factors might explain the association between neighborhood SES and outcomes using mediation proportions, which were estimated by comparing estimates from models with and without the hypothesized mediator.^31^

Because cigarette smoking could confound the health effects of diet and mediate the socioeconomic disparities in health,^32^ we repeated the analyses in never smokers. Statistical analyses were performed using SAS, version 9.4 (SAS Institute Inc), and 2-sided *P* < .05 indicated statistical significance.

## Results

The study included 152 192 health professionals for major CVD analysis (mean [SD] age, 52.0 [8.7] years; 125 959 females [82.8%] and 26 233 males [17.2%]), 151 217 for T2D analysis (mean [SD] age, 52.0 [8.6] years; 125 231 females [82.8%] and 25 986 males [17.2%]), and 141 145 for mortality analysis (mean [SD] age, 51.6 [8.5] years; 117 627 females [83.3%] and 23 518 [16.7%] males). Among all participants for major CVD analysis, 143 409 (94.2%) were White and 8783 (5.8%) were racial or ethnic minority individuals; for T2D analysis, 142 586 (94.3%) were White and 8631 (5.7%) were racial or ethnic minority individuals; and for total mortality analysis, 133 296 (94.4%) were White and 7849 (5.6%) were racial or ethnic minority individuals. Individuals in the top quintile of neighborhood SES or dietary pattern score were more likely to engage in physical activity, have a lower BMI, and take multivitamins and were less likely to have prolonged sedentary television-viewing time or to smoke cigarettes (Table and eTable 1 in Supplement 1).

**Table.** Baseline Characteristics of the Study Population for the Incident CVD Analysis^a^

Characteristic	All participants (N = 152 192)	AHEI-2010 quintile		Neighborhood SES score quintile	
		1st (n = 43 786)	5th (n = 22 605)	1st (n = 30 570)	5th (n = 30 882)
Age, mean (SD), y	52.0 (8.7)	50.3 (8.2)	55.0 (8.9)	52.4 (8.9)	52.3 (8.4)
Sex					
Female	125 959 (82.8)	36 606 (83.6)	17 726 (78.4)	24 890 (81.4)	25 387 (82.2)
Male	26 233 (17.2)	7180 (16.4)	4879 (21.6)	5680 (18.6)	5495 (17.8)
AHEI-2010, mean (SD)	45.8 (9.3)	35.1 (3.7)	61.3 (4.6)	43.9 (9.1)	48.6 (9.3)
Neighborhood SES score, mean (SD)^b^	0.1 (3.5)	−0.7 (3.1)	1.1 (3.8)	−4.3 (1.4)	5.3 (2.2)
Currently married	125 761 (82.6)	36 668 (83.7)	18 304 (81.0)	25 654 (83.9)	25 591 (82.9)
Living alone	13 419 (8.8)	3172 (7.2)	2532 (11.2)	2649 (8.7)	2589 (8.4)
Employed full- or part-time	104 769 (68.8)	30 580 (69.8)	15 103 (66.8)	20 767 (67.9)	21 372 (69.2)
Father’s occupation reported as professional or manager^c^	32 485 (26.4)	8185 (23.0)	5423 (31.1)	4703 (19.4)	8752 (35.2)
Mother’s occupation reported as housewife^c^	78 024 (63.4)	22 485 (63.1)	11 196 (64.2)	15 435 (63.6)	15 865 (63.8)
Household income (≥$150 000/y)^d^	18 855 (26.5)	5234 (23.4)	2520 (31.7)	2613 (18.6)	5977 (41.7)
Husband’s educational level high school or less^c^	32 328 (25.8)	11 462 (31.5)	3583 (20.3)	9152 (37.0)	3438 (13.6)
Race and ethnicity					
White	143 409 (94.2)	41 621 (95.1)	21 025 (93.0)	29 076 (95.1)	28 689 (92.9)
Racial or ethnic minority^e^	8783 (5.8)	2165 (4.9)	1580 (7.0)	1494 (4.9)	2193 (7.1)
Physical activity, mean (SD), METS h/wk	20.2 (20.6)	15.2 (16.2)	28.5 (26.0)	19.1 (19.7)	22.2 (21.9)
Current smoker	15 745 (10.3)	6107 (13.9)	1284 (5.7)	3484 (11.4)	2679 (8.7)
Smoking ≥25 pack-years	20 998 (13.8)	6885 (15.7)	2792 (12.4)	4661 (15.2)	3857 (12.5)
BMI, mean (SD)	25.0 (4.5)	25.3 (4.8)	24.4 (3.9)	25.7 (4.8)	24.2 (3.9)
Alcohol intake, mean (SD), g/d	5.9 (9.9)	5.3 (10.2)	6.4 (9.5)	5.2 (10.0)	7.2 (10.1)
Sleep duration ≤6 h/d	65 741 (43.2)	18 380 (42.0)	10 377 (45.9)	13 031 (42.6)	13 099 (42.4)
Sedentary television-viewing time ≥21 h/wk	16 966 (11.1)	5762 (13.2)	2139 (9.5)	3665 (12.0)	3020 (9.8)
Family history					
Cancer	52 062 (34.2)	14 433 (33.0)	7845 (34.7)	10 065 (32.9)	10 830 (35.1)
Diabetes	38 658 (25.4)	11 329 (25.9)	5399 (23.9)	8303 (27.2)	7161 (23.2)
CVD	62 414 (41.0)	17 588 (40.2)	9421 (41.7)	12 620 (41.3)	12 577 (40.7)
Regular aspirin use^f^	34 086 (22.4)	9131 (20.9)	5756 (25.5)	7335 (24.0)	6504 (21.1)
Regular NSAID use^g^	48 317 (31.7)	14 206 (32.4)	6588 (29.1)	9773 (32.0)	9123 (29.5)
Multivitamin use	75 297 (49.5)	19 595 (44.8)	12 518 (55.4)	14 392 (47.1)	15 814 (51.2)
Postmenopausal hormone use (for women)^c^	27 703 (22.0)	6707 (18.3)	4953 (27.9)	5780 (23.2)	5583 (22.0)
Total energy intake, mean (SD), kcal/d	1828 (502)	1855 (478)	1833 (517)	1861 (519)	1797 (487)

Abbreviations: AHEI-2010, Alternative Healthy Eating Index 2010 (which consists of 11 components, scored from 0 [unhealthiest] to 10 [healthiest]); BMI, body mass index (calculated as weight in kilograms divided by height in meters squared); CVD, cardiovascular disease; METS, metabolic equivalent for task score; NHS, Nurses’ Health Study; NSAID, nonsteroidal anti-inflammatory drug; SES, socioeconomic status.

^a^ Data are presented as No. (%) unless indicated otherwise.

^b^ Computed as the sum of 9 *z* score–standardized census tract–level variables selected by principal component analysis as previously reported.^17^

^c^ Available in the NHS and the NHS II.

^d^ Available in the NHS II.

^e^ Includes American Indian or Alaska Native, Asian, Black, Hispanic, and Native Hawaiian or Other Pacific Islander participants.

^f^ Includes participants who took at least 2 tablets of aspirin (325 mg per tablet) per week in the NHS and at least twice per week in the Health Professionals Follow-Up Study and the NHS II.

^g^ Includes participants who took an NSAID at least twice per week.

**Figure 1.**
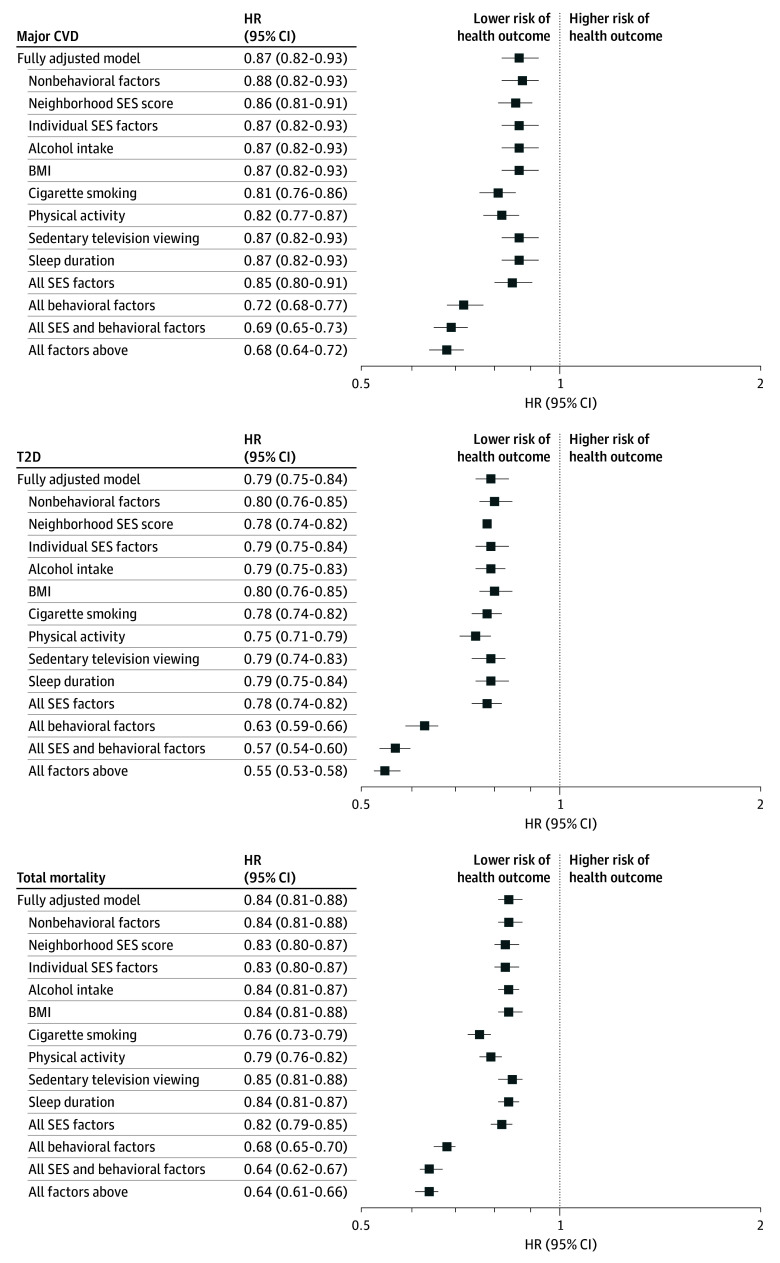
Associations Between Dietary Pattern Score and Primary Outcomes in All Participants The fully adjusted model was adjusted for dietary pattern (Alternative Healthy Eating Index 2010, which consists of 11 components, scored from 0 [unhealthiest] to 10 [healthiest]), total energy intake, nonbehavioral factors (race and ethnicity [White and racial or ethnic minority individuals, including American Indian or Alaska Native, Asian, Black, Hispanic, and Native Hawaiian or Other Pacific Islander]; family history of diabetes, cancer, or cardiovascular disease [CVD]; multivitamin, regular aspirin, or regular nonsteroidal anti-inflammatory drug use; and postmenopausal hormone use for women), neighborhood socioeconomic status (SES) score (computed as the sum of 9 *z* score–standardized census tract–level variables selected by principal component analysis as previously reported^17^), individual SES factors (marital status, husband’s educational level for women, living alone, household income for the Nurses’ Health Study II participants, working status, father’s occupation for women, and mother’s occupation for women), alcohol intake, body mass index (BMI [calculated as weight in kilograms divided by height in meters squared]), cigarette smoking, physical activity, sedentary television-viewing time, and sleep duration. Risk factors were omitted from the fully adjusted model while adjusting for all other variables. Data are presented in eTable 2 in Supplement 1. T2D indicates type 2 diabetes.

During a mean (SD) follow-up of 22.0 (5.2) years, there were 8038 major CVD cases, 11 572 T2D cases, and 19 788 deaths. The Alternative Healthy Eating Index 2010 was inversely associated with risk for major CVD (HR, 0.87 [95% CI, 0.82-0.93), T2D (HR, 0.79 [95% CI, 0.75-0.84]), and total mortality (HR, 0.84 [95% CI, 0.81-0.88]) when adjusted for neighborhood and individual SES and for behavioral and nonbehavioral factors (Figure 1 and eTable 2 in Supplement 1). In general, adjustment for SES minimally altered the associations between diet and health outcomes. Without adjusting for both neighborhood and individual SES factors (while adjusting for behavioral and nonbehavioral variables), the HRs were 0.85 (95% CI, 0.80-0.91) for major CVD, 0.78 (95% CI, 0.74-0.82) for T2D, and 0.82 (95% CI, 0.79-0.85) for total mortality; the corresponding RHRs were 0.98 (95% CI, 0.89-1.07) for major CVD, 0.99 (95% CI, 0.91-1.07) for T2D, and 0.98 (95% CI, 0.92-1.03) for total mortality. In contrast, without adjusting for behavioral factors (while adjusting for SES and nonbehavioral variables), the HRs were 0.72 (95% CI, 0.68-0.77) for major CVD, 0.63 (95% CI, 0.59-0.66) for T2D, and 0.68 (95% CI, 0.65-0.70) for total mortality; the corresponding RHRs were 0.83 (95% CI, 0.76-0.90) for major CVD, 0.80 (95% CI, 0.74-0.86) for T2D, and 0.81 (95% CI, 0.77-0.86) for total mortality. Similarly, the HRs for the association between dietary pattern score and cause-specific mortality showed minimal changes when neighborhood and individual SES factors were omitted but changed modestly when behavioral factors were omitted (eFigure 2 in Supplement 1). Individual SES factors (especially living arrangement and working status) were associated with neurodegenerative disease mortality, with an RHR of 0.95 (95% CI, 0.80-1.12) for SES factors in composite. Among individual factors, cigarette smoking and physical activity altered the associations between diet and disease more substantially than did other factors. We estimated E-values for the diet and disease association as follows: 1.56 for CVD, 1.85 for T2D, and 1.67 for total mortality in all participants and 1.56 for CVD, 1.81 for T2D, and 1.50 for total mortality in never smokers.

**Figure 2.**
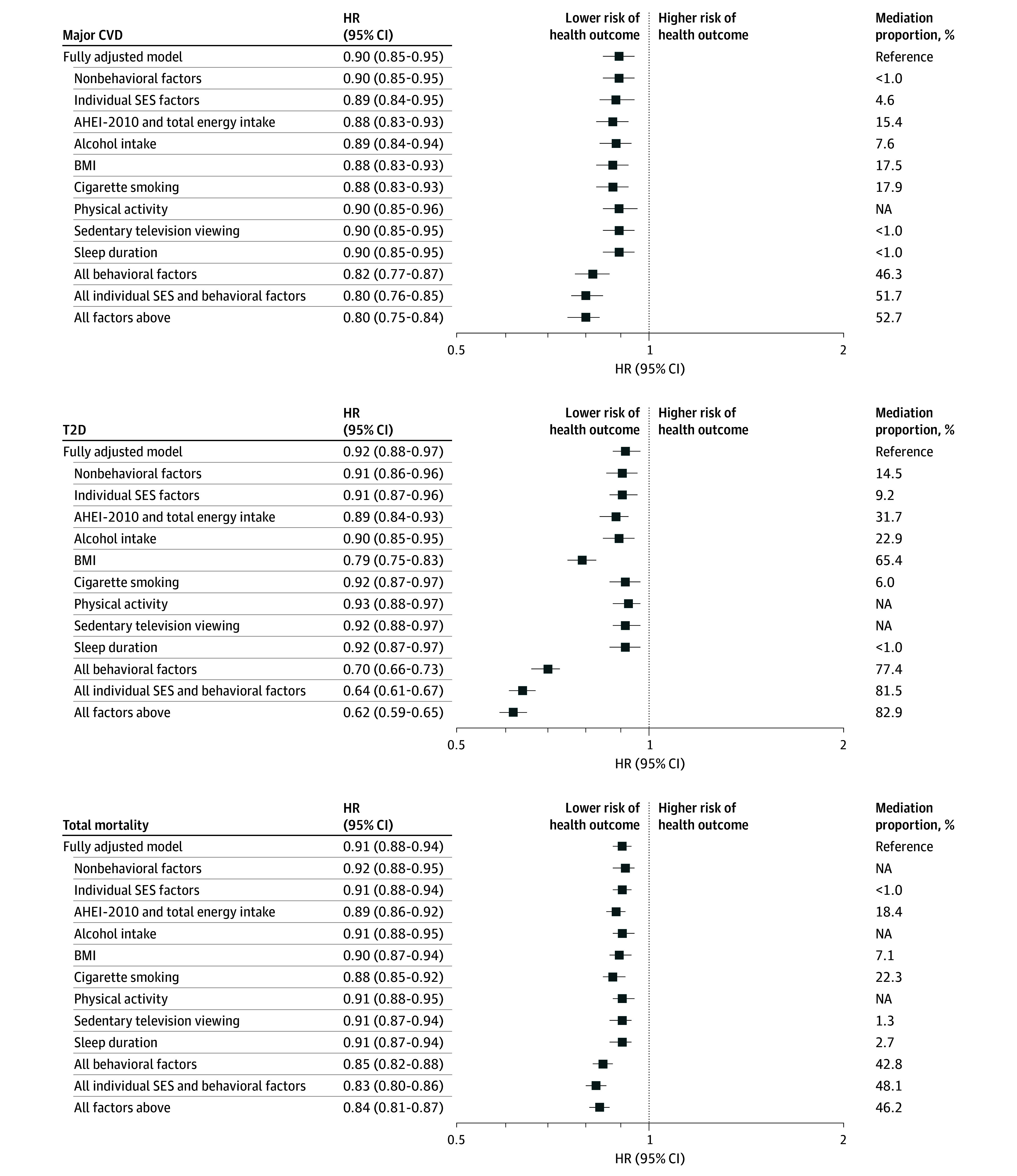
Associations Between Neighborhood Socioeconomic Status (SES) Score and Primary Outcomes in All Participants The fully adjusted model was adjusted for dietary pattern (Alternative Healthy Eating Index 2010 [AHEI-2010], which consists of 11 components, scored from 0 [unhealthiest] to 10 [healthiest]), total energy intake, nonbehavioral factors (race and ethnicity [White and racial or ethnic minority individuals, including American Indian or Alaska Native, Asian, Black, Hispanic, and Native Hawaiian or Other Pacific Islander participants]; family history of diabetes, cancer, or cardiovascular disease [CVD]; multivitamin, regular aspirin, and regular nonsteroidal anti-inflammatory drug use; and postmenopausal hormone use for women), neighborhood SES score (computed as the sum of 9 *z* score–standardized census tract–level variables selected by principal component analysis as previously reported^17^), individual SES factors (marital status, husband’s educational level for women, living alone, household income for the Nurses’ Health Study II participants, working status, father’s occupation for women, and mother’s occupation for women), alcohol intake, body mass index (BMI [calculated as weight in kilograms divided by height in meters squared]), cigarette smoking, physical activity, sedentary television-viewing time, and sleep duration. Risk factors were omitted from the fully adjusted model while adjusting for all other variables. Hazard ratios (HRs) per 10th to 90th percentile are shown. Mediation proportions were not available (NA) when associations were attenuated without adjusting for hypothesized mediators compared with the fully adjusted model (eTable 3 in Supplement 1); proportions given as <1% are presented as such because they cannot be calculated reliably. T2D indicates type 2 diabetes.

A higher neighborhood SES score was associated with a lower risk for major CVD (HR, 0.90 [95% CI, 0.85-0.95), T2D (HR, 0.92 [95% CI, 0.88-0.97]), and total mortality (HR, 0.91 [95% CI], 0.88-0.94) when adjusted for individual SES and behavioral and nonbehavioral factors (Figure 2 and eTable 3 in Supplement 1). Without adjustment for behavioral factors, the HRs changed to 0.82 (95% CI, 0.77-0.87) for major CVD, 0.70 (95% CI, 0.66-0.73) for T2D, and 0.85 (95% CI, 0.82-0.88) for total mortality. The mediation proportions estimated for behavioral factors in composite were 46.3% (95% CI, 32.5%-60.6%) of the association with major CVD, 77.4% (95% CI, 64.5%-86.6%) with T2D, and 42.8% (95% CI, 32.9%–53.3%) with total mortality. Dietary pattern (and total energy intake) accounted for 15.4% (95% CI, 7.9%-27.7%) of the association with major CVD, 31.7% (95% CI, 19.1%-47.7%) with T2D, and 18.4% (95% CI, 12.2%-26.8%) with total mortality. Similarly, behavioral factors accounted for a large proportion of the associations between neighborhood SES score and cause-specific mortality (11.2% for mortality from neurodegenerative disease to 75.3% for cancer mortality) (eFigure 3 in Supplement 1). Among individual factors, BMI mediated a larger proportion of the association between diet and T2D than other factors.

The inverse associations of dietary pattern and neighborhood SES with health outcomes remained in participants who had never smoked cigarettes (eFigures 4 and 5 in Supplement 1). The extent to which neighborhood and individual SES altered estimates of dietary patterns in never smokers was comparable with that for all participants. The associations between neighborhood SES and outcomes accounted for by behavioral factors were also comparable with those in all participants.

## Discussion

Observational studies with long-term follow-up have documented robust and reproducible associations of dietary patterns with many important health outcomes. Although potentially confounding variables have been extensively accounted for in most recent studies, concerns have been raised about residual confounding by SES.^3^ This large prospective cohort study of health professionals found that adjustment for neighborhood and individual SES factors minimally altered the associations between dietary pattern and long-term health, while behavioral variables accounted for a large proportion of the associations between neighborhood SES and health outcomes.

Despite its deterministic role in health, SES is not always measured in observational studies focusing on behavioral factors, particularly diet. Multiple SES-associated factors could affect diet quality and consequently confound the diet and health association. Healthy foods tend to be more affordable and accessible in higher-income neighborhoods, while advertisements for cheaper, unhealthy foods are often targeted at individuals with lower SES residing in lower-income areas.^5,33,34^ Individual SES factors, such as educational level, occupation, and income, may also shape diet quality by possibly affecting an individual’s cooking skills, food literacy, attitudes, and capacity to prioritize a healthy diet.^35^ In this study population with relatively homogeneous SES, we found minimal changes in associations of dietary patterns with health outcomes without adjusting for both individual and neighborhood SES factors. The robust dietary associations and relatively large E-values suggest that substantial residual confounding by SES was unlikely, implying that promoting a healthy diet may provide additional health benefits beyond reducing socioeconomic disparities.

Differences in measuring SES and modeling behavioral variables may limit the direct comparability of our study with previous studies.^36,37,38,39,40^ Despite this, our findings are generally consistent with existing studies on specific diseases or total mortality, which reported that behavioral factors accounted for 16% to 33% of the health disparities associated with individual SES.^41^ A recent large-scale study reported that lifestyle factors assessed at 1 time point (including cigarette smoking, alcohol consumption, physical activity, and dietary quality) compositely accounted for only 12.3% of associations between individual SES and total mortality in the US population and 4.0% in the UK population.^39^ In contrast, we found that behavioral factors accounted for approximately 46.3% of associations for major CVD, 77.4% for T2D, and 42.8% for total mortality. The difference could have resulted from smaller SES variations in health professionals than the general US population or distinct features of neighborhood compared with individual SES measures. The increase in the explanatory role may also be related to a better assessment of neighborhood SES and health behaviors with repeated measures.^36,40,42^

Health behaviors may have different roles in explaining socioeconomic disparities with different outcomes. In line with our estimates, DeVille et al^17^ found that diet, physical activity, and cigarette smoking jointly accounted for 35.0% to 38.9% of the associations between neighborhood SES and total mortality in nurses. Behavioral factors accounted for greater proportions of the associations of neighborhood SES with incident T2D than with major CVD or total mortality, which parallels the associations of diet with T2D compared with other outcomes. Pathways independent of health behaviors, such as lack of insurance or access to proper treatment, may have a larger impact on mortality than on incidence. Despite its decreasing prevalence, cigarette smoking is still seen as a typical unhealthy behavior associated with social patterning.^8^ Cigarette smoking, in addition to its association with SES, could complicate epidemiologic studies on diet owing to its complex association with body weight. Similar findings in our study among never smokers suggest that benefits of healthy behaviors are independent of smoking.

Our study underscores the importance of dietary interventions in addressing health disparities associated with neighborhood SES. Among different health behaviors, dietary factors and BMI accounted for most of the associations between neighborhood SES and outcomes, particularly T2D. Diet may have been underappreciated in a prior study that reached a different conclusion on the joint outcome of health behaviors, as all behaviors were assumed to be equal, and BMI was not incorporated.^39^ Also, the prior study relied on 24-hour recalls,^39^ whereas our study used repeated food-frequency questionnaires for dietary assessment, providing better reflection of long-term dietary intake. For example, another study reported an HR of 0.96 (95% CI, 0.91-1.00) comparing extreme levels of dietary index with all-cause mortality in the UK Biobank,^43^ whereas we observed an HR of 0.84 (95% CI, 0.81-0.88). Measurement errors of diet would have led to underestimation of its health outcome, despite measurement error being decreased using repeated assessments.

### Strengths and Limitations

Major strengths of this study are the rich information on SES and behavioral factors from 3 large cohorts with a long follow-up period. The unique characteristics of the study population provide an opportunity to disentangle confounding of SES factors from the diet and disease association. Unlike prior studies that mostly focused on individual SES and mortality, this study demonstrates that health behaviors accounted for a large portion of incident major chronic diseases associated with neighborhood SES.

Several limitations should also be considered. First, the generalizability of the results might be limited, as the population primarily comprised health professionals with a relatively large proportion of women. Health professionals had better individual SES levels than the general population, but their neighborhood SES levels were comparable.^44,45^ A more homogeneous SES, possibly leading to less residual confounding, may have been an advantage for disentangling the independent association of diet. Second, socioeconomic disparities measured by neighborhood SES could be different from those by individual SES. However, individual-level measures are possibly affected by neighborhood factors, and there is no consensus on which individual-level measures are superior. In turn, a composite score of neighborhood SES factors may have been more useful to characterize the overall SES disparities. Third, reverse causality cannot be ruled out, especially in mortality analyses; we sought to address this by applying a 4-year lag to exposure and covariates. Fourth, although BMI is largely modifiable, as demonstrated by large increases observed in the general US population in recent decades,^46^ it has a genetic component and can be affected by environmental factors such as aggressive marketing and food environment, which are beyond individual control.

## Conclusions

In this prospective cohort study of US health professionals, adjusting for both individual and neighborhood SES minimally altered the associations between diet and long-term health outcomes, suggesting that residual confounding from both neighborhood and individual SES was unlikely to be substantial within this population. In contrast, we found that health behaviors, including dietary pattern, accounted for a large proportion of associations between neighborhood SES and health. Our findings underscore the importance of potentially modifiable health behaviors, particularly high-quality diets, for possibly improving health and reducing socioeconomic disparities.

## Notes

Supplement 1.
eMethods

eReferences
**eFigure 1.** Flowchart of Participants Included in the Incident and Mortality Analyses **eTable 1.** Baseline Characteristics of the Study Population for the Incident Type 2 Diabetes and Mortality Analyses **eTable 2.** Associations Between Dietary Pattern Score and Health Outcomes in All Participants **eFigure 2.** Associations Between the Dietary Pattern Score and Cause-Specific Mortality in All Participants **eTable 3.** Associations Between Neighborhood Socioeconomic Score and Health Outcomes in All Participants **eFigure 3.** Associations Between the Neighborhood Socioeconomic Status Score and Cause-Specific Mortality in All Participants **eFigure 4.** Associations Between the Dietary Pattern Score and Primary Outcomes in Never Smokers **eFigure 5.** Associations Between the Neighborhood Socioeconomic Status Score and Primary Outcomes in Never Smokers

Supplement 2.
Data Sharing Statement
