# Supplementary material for: Socioeconomic Status, Diet, and Behavioral Factors and Cardiometabolic Diseases and Mortality
Source: JAMA Netw Open. 2024 Dec 20;7(12):e2451837. doi: 10.1001/jamanetworkopen.2024.51837 (PMC11662255; doi:10.1001/jamanetworkopen.2024.51837)
Supplement: Supplement 1. — eMethods eReferences eFigure 1. Flowchart of Participants Included in the Incident and Mortality Analyses eTable 1. Baseline Characteristics of the Study Population for the Incident Type 2 Diabetes and Mortality Analyses eTable 2. Associations Between Dietary Pattern Score and Health Outcomes in All Participants eFigure 2. Associations Between the Dietary Pattern Score and Cause-Specific Mortality in All Participants eTable 3. Associations Between Neighborhood Socioeconomic Score and Health Outcomes in All Participants eFigure 3. Associations Between the Neighborhood Socioeconomic Status Score and Cause-Specific Mortality in All Participants eFigure 4. Associations Between the Dietary Pattern Score and Primary Outcomes in Never Smokers eFigure 5. Associations Between the Neighborhood Socioeconomic Status Score and Primary Outcomes in Never Smokers [file jamanetwopen-e2451837-s001.pdf]

Supplemental Online Content

Wang P, Gao X, Willett WC, Giovannucci EL. Socioeconomic status, diet, and behavioral factors and cardiometabolic diseases and mortality. *JAMA Netw Open*. 2024;7(12):e2451837. doi:10.1001/jamanetworkopen.2024.51837

eMethods
eReferences
eFigure 1. Flowchart of Participants Included in the Incident and Mortality Analyses
eTable 1. Baseline Characteristics of the Study Population for the Incident Type 2 Diabetes and Mortality Analyses
eTable 2. Associations Between Dietary Pattern Score and Health Outcomes in All Participants
eFigure 2. Associations Between the Dietary Pattern Score and Cause-Specific Mortality in All Participants
eTable 3. Associations Between Neighborhood Socioeconomic Score and Health Outcomes in All Participants
eFigure 3. Associations Between the Neighborhood Socioeconomic Status Score and Cause-Specific Mortality in All Participants
eFigure 4. Associations Between the Dietary Pattern Score and Primary Outcomes in Never Smokers
eFigure 5. Associations Between the Neighborhood Socioeconomic Status Score and Primary Outcomes in Never Smokers

This supplemental material has been provided by the authors to give readers additional information about their work.

© 2024 Wang P et al. *JAMA Network Open*.

eMethods

Assessment of socioeconomic status

The neighborhood SES was assessed as previously reported.[1] Briefly, census-tract variables were extracted and updated by mapping Census data from the Neighborhood Change Database to participants' residential addresses. The neighborhood SES score was computed as the sum of nine z-standardized census-tract level variables selected by principal component analysis, including median household income, median home value, percent with a college degree or more, percent of household receiving interest or dividends (reversed by subtracting from one), percent of occupied housing units, percent of non-Hispanic White, percent of non-Hispanic Black, percent of foreign-born, and percent unemployment.

Participants from all three cohorts answered biennial questionnaires on their current marital status (married, divorced or separated, widowed, or never married), living arrangement (alone, with partner, with other family, nursing home, or other), and working status (full-time, part-time, retired, or disabled). Nurses also provided information on their parents' occupations (professional, executive or manager, sales or clerical worker, mechanic worker, machine operator or driver, service work, laborer, farming, military, or did not work) and husband's highest education level (less than high school, high school, 2-year college, 4-year college, or graduate school). Participants from the NHS II provided additional information on household income (< $15 000, $15 000–<$20 000, $20 000–<$30 000, $30 000–<$40 000, $40 000–<$50 000, $40 000–<$50 000, $50 000–<$75 000, $75 000–<$100 000, $100 000–<$150 000, or ≥$150 000).

Assessment of other factors

Participants reported their smoking behavior, including current smoking status, daily cigarette consumption, and age at which they began or stopped smoking. We calculated pack-years of smoking and duration of smoking cessation. Total metabolic equivalent-hours/week of physical activity was derived based on questions on types and duration of time spent for each type of activity, which were collected starting from 1986 in the NHS, 1989 in the NHS II, and 1986 in the HPFS. We included sedentary TV viewing time because a study in present cohorts showed that sedentary TV viewing was a stronger predictor of adverse health outcomes than other sedentary behaviors.[2] Sedentary TV viewing time was assessed staring from 1992 in the NHS, 1991 in the NHS II, and 1988 in the HPFS. Participants were asked to indicate how many hours of actual sleep they have in a 24-hour period starting since 1986 in the NHS, 2001 in the NHS II, and 1987 in the HPFS. Frequency of aspirin use was reported starting from 1984 in the NHS, 1989 in the NHS II, and 1986 in the HPFS. Frequency of nonsteroidal anti-inflammatory drug use was reported starting from 1990 in the NHS, 1989 in the NHS II, and 1986 in the HPFS. Regular aspirin use was defined as taking at least two tablets of aspirin (325 mg per tablet) per week in the NHS and at least twice a week in the HPFS and NHS II. Regular use of nonsteroidal anti-inflammatory drug was defined as taking at least twice a week.

Outcome ascertainment

Deaths were classified into different causes using the International Classification of Diseases (ICD)-8 and ICD-9.[3] We considered major causes of death including cardiovascular disease (ICD-8: 390–458; ICD-9: 390–459), cancer (ICD-8: 140–209; ICD-9: 140–208), respiratory disease (ICD-8: 460–519; ICD-9: 460–519) and neurodegenerative disease (ICD-8: 290, 340, 342, and 348; ICD-9: 290, 332, 335, 340, 342, and 348).

eReferences

1. DeVille NV, Iyer HS, Holland I, et al. Neighborhood socioeconomic status and mortality in the nurses' health study (NHS) and the nurses' health study II (NHSII). *Environ Epidemiol*. Feb 2023;7(1):e235. doi:10.1097/EE9.0000000000000235
2. Hu FB, Li TY, Colditz GA, Willett WC, Manson JE. Television watching and other sedentary behaviors in relation to risk of obesity and type 2 diabetes mellitus in women. *JAMA*. Apr 9 2003;289(14):1785-91. doi:10.1001/jama.289.14.1785
3. Shan Z, Wang F, Li Y, et al. Healthy Eating Patterns and Risk of Total and Cause-Specific Mortality. *JAMA Intern Med*. Feb 1 2023;183(2):142-153. doi:10.1001/jamainternmed.2022.6117

eFigure 1. Flowchart of Participants Included in the Incident and Mortality Analyses

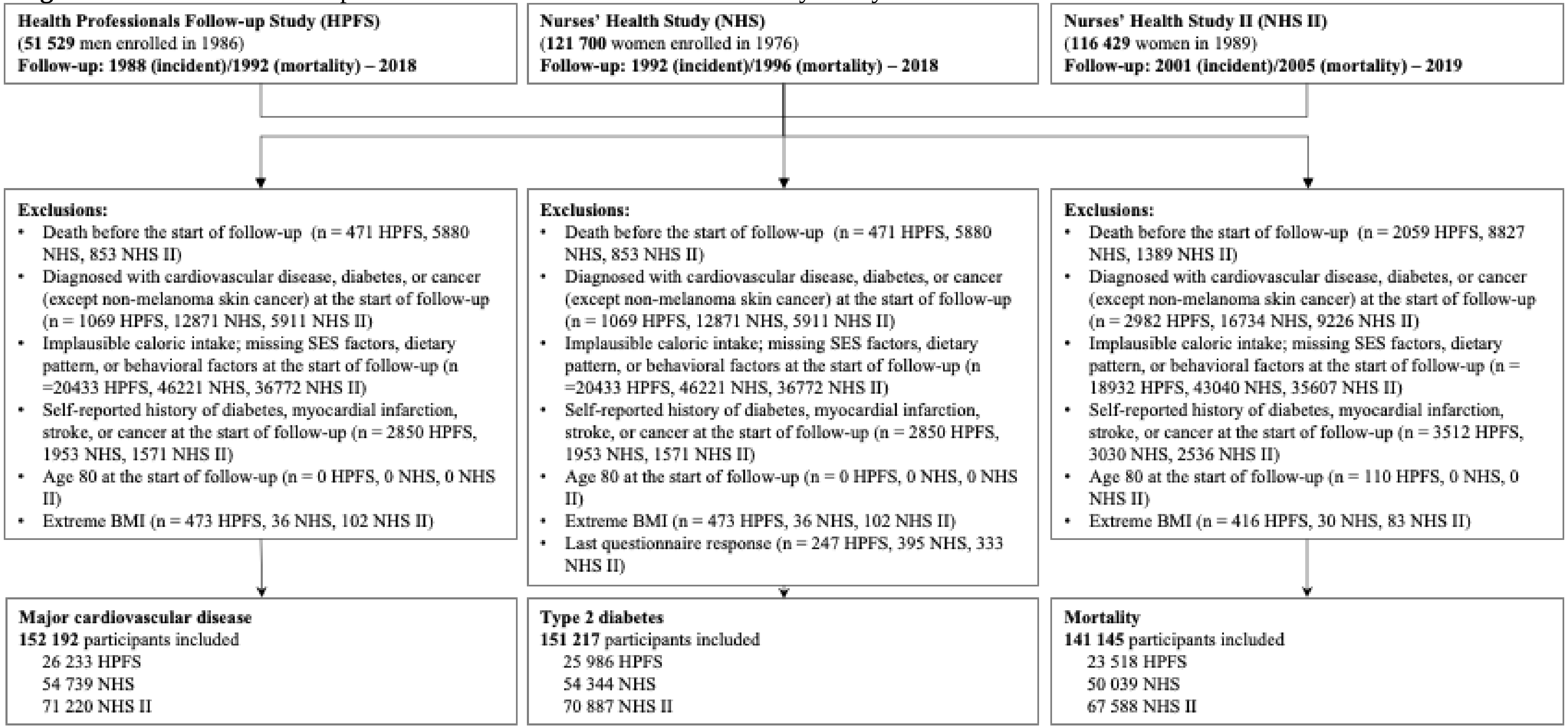

BMI, body mass index; HPFS, Health Professionals Follow-up Study; NHS, Nurses' Health Study; SES, socioeconomic status.

© 2024 Wang P et al. *JAMA Network Open*.

eTable 1. Baseline Characteristics of the Study Population for the Incident Type 2 Diabetes and Mortality Analyses[a]

	Type 2 diabetes analysis				
		AHEI-2010		Neighborhood SES score	
	All participants (N = 151 217)	Quintile 1 (N = 42 444)	Quintile 5 (N = 23 066)	Quintile 1 (N = 30 604)	Quintile 5 (N = 30 286)
Age, year	52.0 (8.6)	50.4 (8.2)	54.7 (8.9)	52.3 (8.9)	52.3 (8.4)
Male	25 986 (17.2)	7014 (16.5)	4871 (21.1)	5631 (18.4)	5367 (17.7)
Female	125 231 (82.8)	35 430 (83.5)	18 195 (78.9)	24 973 (81.6)	24 919 (82.3)
Alternative Healthy Eating Index-2010	45.8 (9.3)	35.0 (3.7)	61.1 (4.6)	43.9 (9.1)	48.6 (9.3)
Neighborhood SES score[b]	0.1 (3.5)	-0.7 (3.1)	1.1 (3.8)	-4.3 (1.4)	5.3 (2.2)
Currently married	125 029 (82.7)	35 563 (83.8)	18 662 (80.9)	25 697 (84.0)	25 120 (82.9)
Living alone	13 286 (8.8)	3075 (7.2)	2552 (11.1)	2634 (8.6)	2533 (8.4)
Working status (full/part-time)	104 250 (68.9)	29 708 (70.0)	15 485 (67.1)	20 838 (68.1)	20 973 (69.2)
Father's occupation (professional/manager)[c]	32 370 (26.4)	7905 (22.9)	5588 (31.2)	4724 (19.4)	8632 (35.3)
Mother's occupation (housewife)[c]	77 775 (63.4)	21 847 (63.2)	11 507 (64.2)	15 526 (63.6)	15 616 (63.8)
Household income (≥$150,000)[d]	18 748 (26.4)	4966 (23.3)	2712 (31.7)	2627 (18.6)	5864 (41.8)
Husband's education (high school or less)[c]	32 114 (25.8)	11 226 (31.9)	3587 (19.8)	9161 (36.9)	3369 (13.6)
Race and ethnicity					
Non-Hispanic White	142 586 (94.3)	40 355 (95.1)	21 476 (93.1)	29 132 (95.2)	28 139 (92.9)
Others[e]	8631 (5.7)	2089 (4.9)	1590 (6.9)	1472 (4.8)	2147 (7.1)
Physical activity, METS-h/week	20.2 (20.6)	15.1 (16.1)	28.5 (25.9)	19.1 (19.7)	22.2 (21.8)
Current smoking	15 513 (10.3)	5925 (14.0)	1314 (5.7)	3462 (11.3)	2593 (8.6)
Smoking ≥25 pack-years	20 661 (13.7)	6690 (15.8)	2766 (12.0)\	4602 (15.0)	3761 (12.4)
Body mass index, kg/m^2	25.0 (4.5)	25.3 (4.8)	24.4 (3.9)	25.7 (4.8)	24.2 (3.9)
Alcohol intake, g/day	5.9 (9.9)	5.4 (10.2)	6.3 (9.4)	5.2 (10.0)	7.2 (10.1)
Sleep duration (≤6 h/day)	65 281 (43.2)	17 944 (42.3)	10 442 (45.3)	13 059 (42.7)	12 852 (42.4)
Sedentary TV viewing time (≥21 h/week)	16 796 (11.1)	5599 (13.2)	2132 (9.2)	3662 (12)	2947 (9.7)
Family history of cancer	51 761 (34.2)	13 996 (33.0)	8004 (34.7)	10 085 (33.0)	10 657 (35.2)
Family history of diabetes	38 460 (25.4)	10 968 (25.8)	5556 (24.1)	8332 (27.2)	7027 (23.2)
Family history of CVD	61 982 (41.0)	16 986 (40.0)	9644 (41.8)	12 643 (41.3)	12 332 (40.7)
Regular aspirin use[f]	33 808 (22.4)	8919 (21.0)	5784 (25.1)	7305 (23.9)	6366 (21.0)
Regular NSAID use[g]	48 055 (31.8)	13 824 (32.6)	6725 (29.2)	9803 (32.0)	8960 (29.6)
Multivitamin use	74 845 (49.5)	18 905 (44.5)	12 829 (55.6)	14 406 (47.1)	15 495 (51.2)
Postmenopausal hormone use[c]	27 559 (22.0)	6532 (18.4)	4993 (27.4)	5799 (23.2)	5498 (22.1)
Total energy intake, kcal/d	1828 (502)	1854 (477)	1836 (517)	1861 (518)	1797 (487)

© 2024 Wang P et al. *JAMA Network Open*.

	Mortality analysis				
		AHEI-2010		Neighborhood SES score	
	All participants (N = 141 145)	Quintile 1 (N = 38 197)	Quintile 5 (N = 22 435)	Quintile 1 (N = 28 241)	Quintile 5 (N = 28 576)
Age, year	51.6 (8.5)	50.0 (8.0)	54.2 (8.7)	51.9 (8.7)	52.0 (8.2)
Male	23 518 (16.7)	6269 (16.4)	4465 (19.9)	5059 (17.9)	4864 (17.0)
Female	117 627 (83.3)	31 928 (83.6)	17 970 (80.1)	23 182 (82.1)	23 712 (83.0)
Alternative Healthy Eating Index-2010	45.8 (9.3)	34.8 (3.6)	60.8 (4.6)	43.8 (9.1)	48.6 (9.3)
Neighborhood SES score[b]	0.1 (3.5)	-0.7 (3.1)	1.1 (3.8)	-4.3 (1.4)	5.3 (2.2)
Currently married	116 937 (82.8)	32 115 (84.1)	18 105 (80.7)	23 775 (84.2)	23 716 (83.0)
Living alone	12 155 (8.6)	2687 (7.0)	2476 (11.0)	2384 (8.4)	2354 (8.2)
Working status (full/part-time)	98 036 (69.5)	26 987 (70.7)	15 221 (67.8)	19 415 (68.7)	19 916 (69.7)
Father's occupation (professional/manager)[c]	30 560 (26.6)	7130 (22.9)	5550 (31.4)	4389 (19.4)	8240 (35.5)
Mother's occupation (housewife)[c]	72 955 (63.5)	19 660 (63.3)	11 345 (64.2)	14 415 (63.7)	14 817 (63.8)
Household income (≥$150,000)[d]	17 999 (26.6)	4513 (23.4)	2810 (31.9)	2498 (18.8)	5678 (42.0)
Husband's education (high school or less)[c]	29 686 (25.4)	10 075 (31.8)	3465 (19.4)	8397 (36.5)	3134 (13.3
Race and ethnicity					
Non-Hispanic White	133 296 (94.4)	36 373 (95.2)	20 918 (93.2)	26 921 (95.3)	26 578 (93.0)
Others[e]	7849 (5.6)	1824 (4.8)	1517 (6.8)	1320 (4.7)	1998 (7.0)
Physical activity, METS-h/week	20.3 (20.6)	15.1 (16.1)	28.6 (26.0)	19.2 (19.7)	22.3 (21.9)
Current smoking	14 206 (10.1)	5289 (13.8)	1284 (5.7)	3119 (11.0)	2425 (8.5)
Smoking ≥25 pack-years	18 297 (13.0)	5809 (15.2)	2523 (11.2)	4033 (14.3)	3378 (11.8)
Body mass index, kg/m^2	24.9 (4.4)	25.1 (4.7)	24.3 (3.9)	25.5 (4.7)	24.1 (3.9)
Alcohol intake, g/day	5.9 (9.7)	5.3 (10.1)	6.3 (9.3)	5.1 (9.8)	7.1 (10.0)
Sleep duration (≤6 h/day)	60 587 (42.9)	16 104 (42.2)	10 079 (44.9)	11 970 (42.4)	12 038 (42.1)
Sedentary TV viewing time (≥21 h/week)	15 171 (10.7)	4915 (12.9)	1982 (8.8)	3273 (11.6)	2687 (9.4)
Family history of cancer	48 148 (34.1)	12 506 (32.7)	7789 (34.7)	9285 (32.9)	10 050 (35.2)
Family history of diabetes	35 414 (25.1)	9700 (25.4)	5338 (23.8)	7573 (26.8)	6556 (22.9)
Family history of CVD	57 519 (40.8)	15 139 (39.6)	9381 (41.8)	11 577 (41.0)	11 562 (40.5)
Regular aspirin use[f]	30 842 (21.9)	7902 (20.7)	5432 (24.2)	6612 (23.4)	5882 (20.6)
Regular NSAID use[g]	44 860 (31.8)	12 383 (32.4)	6606 (29.4)	9053 (32.1)	8452 (29.6)
Multivitamin use	70 095 (49.7)	17 030 (44.6)	12 551 (55.9)	13 361 (47.3)	14 662 (51.3)
Postmenopausal hormone use[c]	25 650 (21.8)	5835 (18.3)	4858 (27.0)	5353 (23.1)	5161 (21.8)
Total energy intake, kcal/d	1828 (500)	1853 (475)	1835 (516)	1861 (517)	1796 (484)

© 2024 Wang P et al. *JAMA Network Open*.

AHEI-2010, Alternative Healthy Eating Index 2010 (which consists of 11 components, scored 1 from 0 [unhealthiest] to 10 [healthiest]); BMI, body mass index (calculated as weight in kilograms divided by height in meters squared); CVD, cardiovascular disease; HPFS, Health Professionals Follow-Up; METS, metabolic equivalent for task score; NHS, Nurses' Health Study; NSAID, nonsteroidal anti-inflammatory drug; SES, socioeconomic status.

[a]Data are presented as No. (%) unless indicated otherwise.

[b]Computed as the sum of 9 *z* score–standardized census tract–level variables selected by principal component analysis as previously reported.

[c]Available in the NHS and the NHS II.

[d]Available in the NHS II.

[e]Includes American Indian or Alaska Native, Asian, Black, Hispanic, and Native Hawaiian or Other Pacific Islander Black, Asian, American Indian, Hawaiian, or Hispanic participants.

[f]Includes participants who took at least two 2 tablets of aspirin (325 mg per tablet) per week in the NHS and at least twice per week in the HPFS and the NHS II.

[g]Includes participants who took an NSAID at least twice per week.

© 2024 Wang P et al. *JAMA Network Open*.

eTable 2. Associations Between Dietary Pattern Score and Health Outcomes in All Participants

	Major CVD		Type 2 diabetes		All-cause mortality	
Cases	8,038		11,572		19,788	
Person-years	2,919,693		2,476,866		2,322,444	
All participants	HR (95% CI)	RHR (95% CI)	HR (95% CI)	RHR (95% CI)	HR (95% CI)	RHR (95% CI)
Fully adjusted model[a]	0.87 (0.82, 0.93)	1 (Ref.)	0.79 (0.75, 0.84)	1 (Ref.)	0.84 (0.81, 0.88)	1 (Ref.)
- Non-behavioral factors[b]	0.88 (0.82, 0.93)	1.01 (0.93, 1.11)	0.80 (0.76, 0.85)	1.01 (0.94, 1.10)	0.84 (0.81, 0.88)	1.00 (0.94, 1.06)
- Neighborhood SES score	0.86 (0.81, 0.91)	0.99 (0.91, 1.08)	0.78 (0.74, 0.82)	0.99 (0.91, 1.07)	0.83 (0.80, 0.87)	0.99 (0.93, 1.05)
- Individual SES factors[c]	0.87 (0.82, 0.93)	1.00 (0.91, 1.09)	0.79 (0.75, 0.84)	1.00 (0.92, 1.08)	0.83 (0.80, 0.87)	0.99 (0.93, 1.05)
- Alcohol intake	0.87 (0.82, 0.93)	1.00 (0.91, 1.09)	0.79 (0.75, 0.83)	1.00 (0.93, 1.08)	0.84 (0.81, 0.87)	1.00 (0.95, 1.06)
- BMI	0.87 (0.82, 0.93)	1.00 (0.91, 1.09)	0.80 (0.76, 0.85)	1.01 (0.94, 1.10)	0.84 (0.81, 0.88)	1.00 (0.94, 1.06)
- Cigarette smoking	0.81 (0.76, 0.86)	0.93 (0.85, 1.02)	0.78 (0.74, 0.82)	0.99 (0.91, 1.07)	0.76 (0.73, 0.79)	0.90 (0.85, 0.96)
- Physical activity	0.82 (0.77, 0.87)	0.94 (0.86, 1.03)	0.75 (0.71, 0.79)	0.95 (0.88, 1.03)	0.79 (0.76, 0.82)	0.94 (0.89, 0.99)
- Sedentary TV viewing time	0.87 (0.82, 0.93)	1.00 (0.91, 1.09)	0.79 (0.74, 0.83)	1.00 (0.92, 1.08)	0.85 (0.81, 0.88)	1.01 (0.95, 1.07)
- Sleep duration	0.87 (0.82, 0.93)	1.00 (0.91, 1.09)	0.79 (0.75, 0.84)	1.00 (0.92, 1.08)	0.84 (0.81, 0.87)	1.00 (0.95, 1.06)
- All SES factors	0.85 (0.80, 0.91)	0.98 (0.89, 1.07)	0.78 (0.74, 0.82)	0.99 (0.91, 1.07)	0.82 (0.79, 0.85)	0.98 (0.92, 1.03)
- All behavioral factors	0.72 (0.68, 0.77)	0.83 (0.76, 0.90)	0.63 (0.59, 0.66)	0.80 (0.74, 0.86)	0.68 (0.65, 0.70)	0.81 (0.77, 0.86)
- All SES and behavioral factors	0.69 (0.65, 0.73)	0.79 (0.73, 0.86)	0.57 (0.54, 0.60)	0.72 (0.67, 0.78)	0.64 (0.62, 0.67)	0.76 (0.72, 0.81)
- All factors above	0.68 (0.64, 0.72)	0.78 (0.72, 0.85)	0.55 (0.53, 0.58)	0.70 (0.65, 0.75)	0.64 (0.61, 0.66)	0.76 (0.72, 0.81)

Hazard ratio and 95% confidence intervals for every 10th–90th percentile increment of dietary pattern score are shown. AHEI-2010, Alternative Healthy Eating Index-2010; BMI, body mass index; CI, confidence interval; HR, hazard ratio; RHR, ratio of hazard ratio; SES, socioeconomic status.

[a]Analyses were stratified by age, calendar year, and cohort. Fully adjusted model was adjusted for dietary pattern (AHEI-2010), total energy intake, non-behavioral factors (race, family history of diabetes, family history of cancer, family history of cardiovascular disease, multivitamin use, regular aspirin use, regular non-steroidal anti-inflammatory drugs use, and postmenopausal hormone use for women), neighborhood SES score, individual SES factors, alcohol intake, BMI, cigarette smoking, physical activity, sedentary TV viewing time, and sleep duration.

[b]Non-behavioral risk factors included race, family history of diabetes, family history of cancer, family history of cardiovascular disease, multivitamin use, regular aspirin use, regular non-steroidal anti-inflammatory drugs use, and postmenopausal hormone use for women.

[c]Individual SES factors included marital status, husband's education for women, live alone, household income for NHSII participants, working status, father's occupation for women, and mother's occupation for women.

© 2024 Wang P et al. *JAMA Network Open*.

eFigure 2. Associations Between the Dietary Pattern Score and Cause-Specific Mortality in All Participants

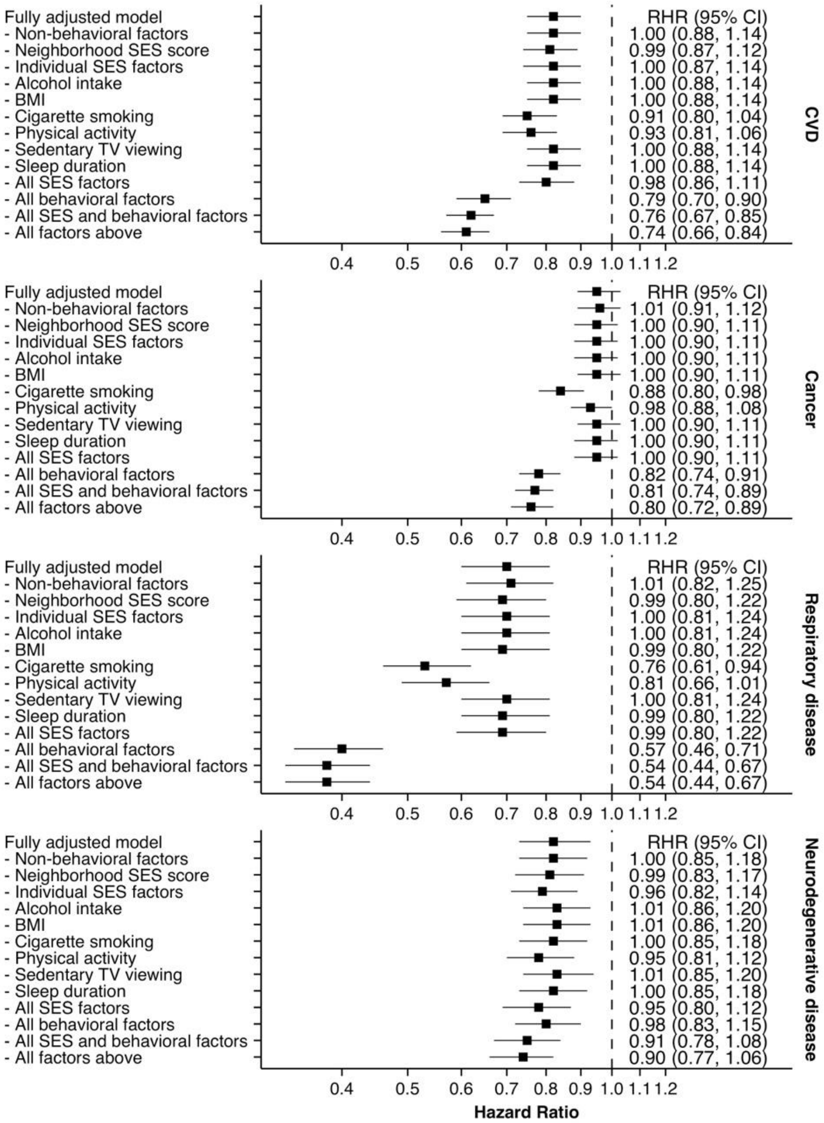

Hazard ratio and 95% confidence intervals for every 10th–90th percentile increment of dietary pattern score are shown on log scales. All analyses were stratified by age, calendar year, and cohort. Fully adjusted model was adjusted for dietary pattern (AHEI-2010), total energy intake, non-behavioral factors (race and ethnicity, family history of diabetes, family history of cancer, family history of cardiovascular disease, multivitamin use, regular aspirin use, regular non-steroidal anti-inflammatory drugs use, and postmenopausal hormone use for women), neighborhood SES score, individual SES factors (marital status, husband's education for women, live alone, household income for NHSII participants, working status, father's occupation for women, and mother's occupation for women), alcohol intake, BMI, cigarette smoking, physical activity, sedentary TV viewing time, and sleep duration. Risk factors were omitted from the fully adjusted model while adjusting for all other variables. The ratio of hazard ratios (RHRs) represents the change in hazard ratios from the multivariable-adjusted model without the specific factor compared with that from the fully adjusted model. AHEI-2010, Alternative Healthy Eating Index-2010; BMI, body mass index; CI, confidence interval; SES, socioeconomic status; RHR, ratio of hazard ratio.

© 2024 Wang P et al. *JAMA Network Open*.

eTable 3. Associations Between Neighborhood Socioeconomic Score and Health Outcomes in All Participants

	Major CVD		Type 2 diabetes		All-cause mortality	
Cases	8,038		11,572		19,788	
Person-years	2,919,693		2,476,866		2,322,444	
All participants	HR (95% CI)	Mediation (%)	HR (95% CI)	Mediation (%)	HR (95% CI)	Mediation (%)
Fully adjusted model[a]	0.90 (0.85, 0.95)		0.92 (0.88, 0.97)		0.91 (0.88, 0.94)	
- Non-behavioral factors[b]	0.90 (0.85, 0.95)	<1	0.91 (0.86, 0.96)	14.5 (7.3, 26.7)	0.92 (0.88, 0.95)	NA
- Individual SES factors[c]	0.89 (0.84, 0.95)	4.6 (0.6, 27.4)	0.91 (0.87, 0.96)	9.2 (2.0, 32.9)	0.91 (0.88, 0.94)	<1
- AHEI-2010 and total energy intake	0.88 (0.83, 0.93)	15.4 (7.9, 27.7)	0.89 (0.84, 0.93)	31.7 (19.1, 47.7)	0.89 (0.86, 0.92)	18.4 (12.2, 26.8)
- Alcohol intake	0.89 (0.84, 0.94)	7.6 (3.8, 14.4)	0.90 (0.85, 0.95)	22.9 (13.4, 36.3)	0.91 (0.88, 0.95)	NA
- BMI	0.88 (0.83, 0.93)	17.5 (10.7, 27.5)	0.79 (0.75, 0.83)	65.4 (50.2, 78.0)	0.90 (0.87, 0.94)	7.1 (4.3, 11.4)
- Cigarette smoking	0.88 (0.83, 0.93)	17.9 (11.0, 27.8)	0.92 (0.87, 0.97)	6.0 (2.8, 12.3)	0.88 (0.85, 0.92)	22.3 (15.8, 30.5)
- Physical activity	0.90 (0.85, 0.96)	NA	0.93 (0.88, 0.97)	NA	0.91 (0.88, 0.95)	NA
- Sedentary TV viewing time	0.90 (0.85, 0.95)	<1	0.92 (0.88, 0.97)	NA	0.91 (0.87, 0.94)	1.3 (0.6, 2.5)
- Sleep duration	0.90 (0.85, 0.95)	<1	0.92 (0.87, 0.97)	<1	0.91 (0.87, 0.94)	2.7 (1.3, 5.4)
- All behavioral factors	0.82 (0.77, 0.87)	46.3 (32.5, 60.6)	0.70 (0.66, 0.73)	77.4 (64.5, 86.6)	0.85 (0.82, 0.88)	42.8 (32.9, 53.3)
- All individual SES and behavioral factors	0.80 (0.76, 0.85)	51.7 (37.1, 66.0)	0.64 (0.61, 0.67)	81.5 (69.9, 89.3)	0.83 (0.80, 0.86)	48.1 (37.5, 58.9)
- All factors above	0.80 (0.75, 0.84)	52.7 (38.0, 66.9)	0.62 (0.59, 0.65)	82.9 (72.0, 90.2)	0.84 (0.81, 0.87)	46.2 (35.5, 57.2)

Hazard ratio and 95% confidence intervals for every 10th–90th percentile increment of neighborhood socioeconomic status score are shown. Mediation proportions were not available when associations were attenuated without adjusting for hypothesized mediators compared to the fully adjusted model. AHEI-2010, Alternative Healthy Eating Index-2010; BMI, body mass index; CI, confidence interval; HR, hazard ratio; NA, not available; SES, socioeconomic status.

[a]Analyses were stratified by age, calendar year, and cohort. Fully adjusted model was adjusted for dietary pattern (AHEI-2010), total energy intake, non-behavioral factors (race, family history of diabetes, family history of cancer, family history of cardiovascular disease, multivitamin use, regular aspirin use, regular non-steroidal anti-inflammatory drugs use, and postmenopausal hormone use for women), neighborhood SES score, individual SES factors, alcohol intake, BMI, cigarette smoking, physical activity, sedentary TV viewing time, and sleep duration.

[b]Non-behavioral risk factors included race, family history of diabetes, family history of cancer, family history of cardiovascular disease, multivitamin use, regular aspirin use, regular non-steroidal anti-inflammatory drugs use, and postmenopausal hormone use for women.

[c]Individual SES factors included marital status, husband's education for women, live alone, household income for NHSII participants, working status, father's occupation for women, and mother's occupation for women.

© 2024 Wang P et al. *JAMA Network Open*.

eFigure 3. Associations Between the Neighborhood Socioeconomic Status Score and Cause-Specific Mortality in All Participants

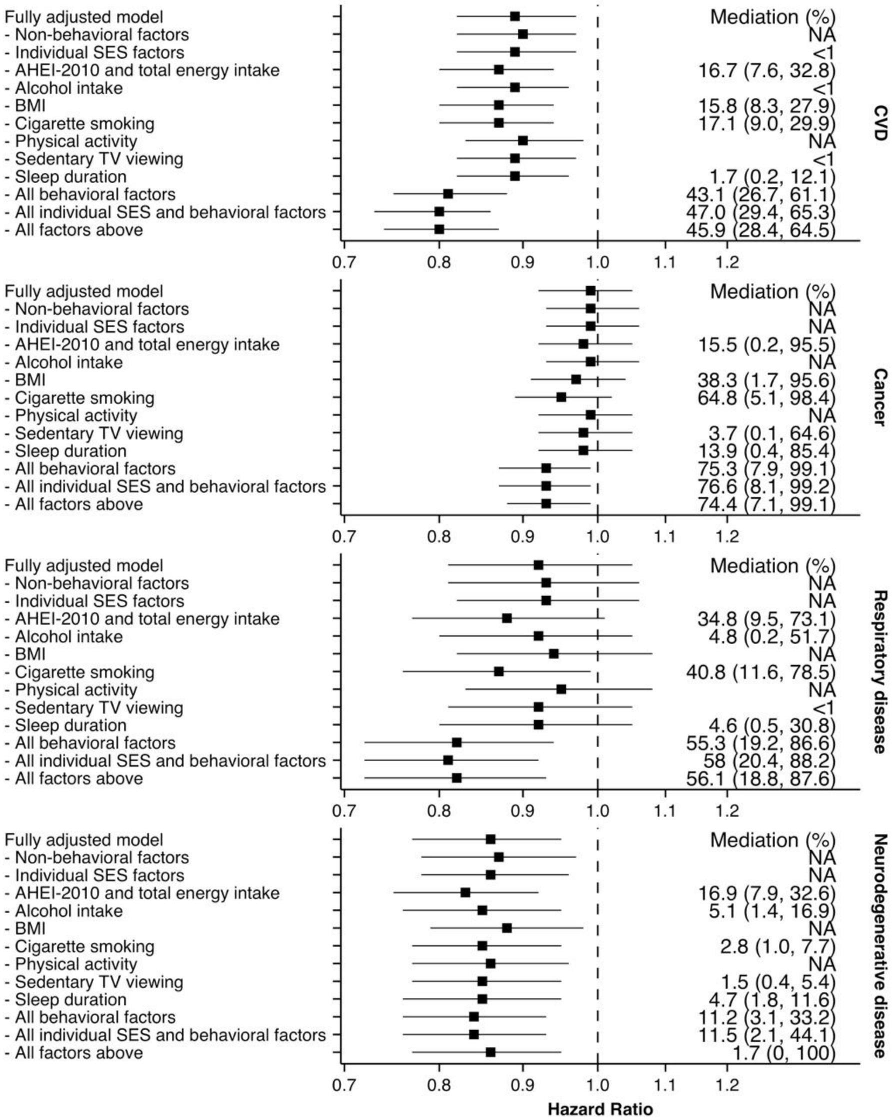

Hazard ratio and 95% confidence intervals for every 10th–90th percentile increment of neighborhood socioeconomic status score are shown on log scales. All analyses were stratified by age, calendar year, and cohort. Fully adjusted model was adjusted for dietary pattern (AHEI-2010), total energy intake, non-behavioral factors (race and ethnicity, family history of diabetes, family history of cancer, family history of cardiovascular disease, multivitamin use, regular aspirin use, regular non-steroidal anti-inflammatory drugs use, and postmenopausal hormone use for women), neighborhood SES score, individual SES factors (marital status, husband's education for women, live alone, household income for NHSII participants, working status, father's occupation for women, and mother's occupation for women), alcohol intake, BMI, cigarette smoking, physical activity, sedentary TV viewing time, and sleep duration. Risk factors were omitted from the fully adjusted model while adjusting for all other variables. Mediation proportions were not available when associations were attenuated without adjusting for hypothesized mediators compared to the fully adjusted model. AHEI-2010, Alternative Healthy Eating Index-2010; BMI, body mass index; CI, confidence interval; NA, not available; SES, socioeconomic status.

© 2024 Wang P et al. *JAMA Network Open*.

eFigure 4. Associations Between the Dietary Pattern Score and Primary Outcomes in Never Smokers

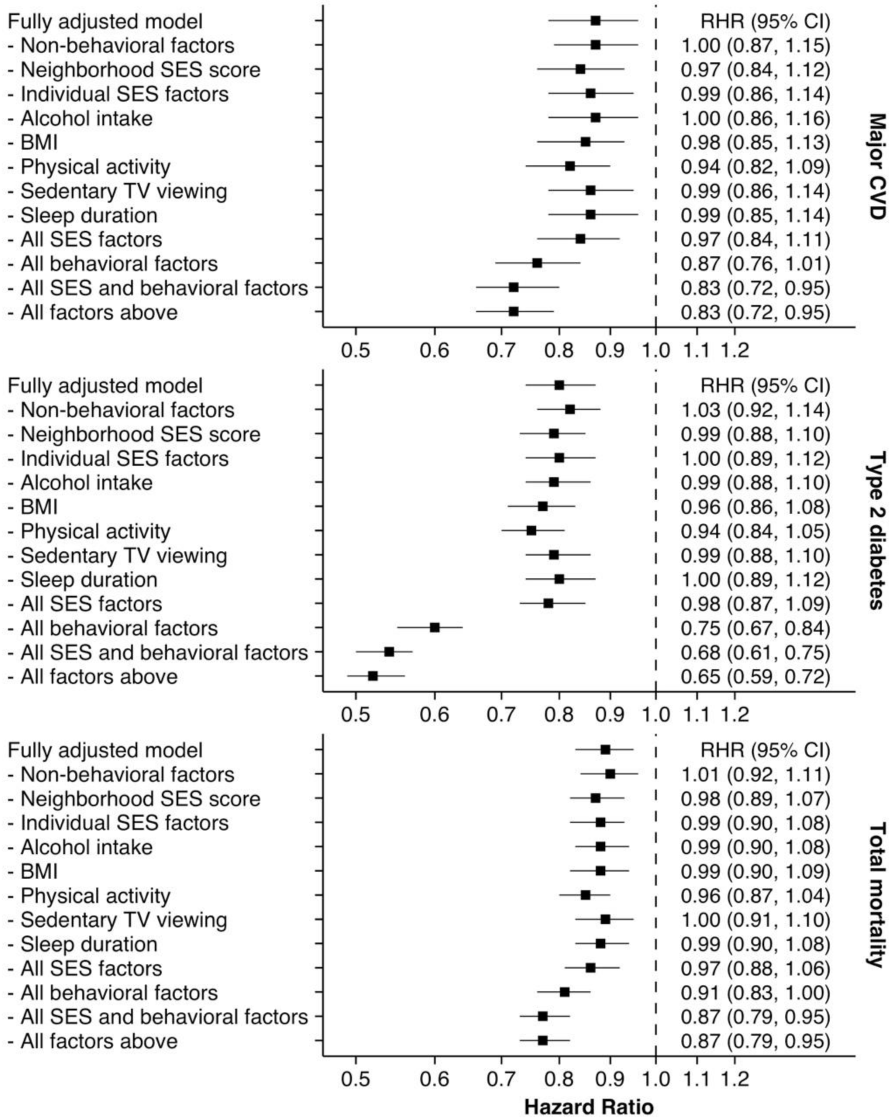

Hazard ratio and 95% confidence intervals for every 10th–90th percentile increment of dietary pattern score are shown on log scales. All analyses were stratified by age, calendar year, and cohort. Fully adjusted model was adjusted for dietary pattern (AHEI-2010), total energy intake, non-behavioral factors (race and ethnicity, family history of diabetes, family history of cancer, family history of cardiovascular disease, multivitamin use, regular aspirin use, regular non-steroidal anti-inflammatory drugs use, and postmenopausal hormone use for women), neighborhood SES score, individual SES factors (marital status, husband's education for women, live alone, household income for NHSII participants, working status, father's occupation for women, and mother's occupation for women), alcohol intake, BMI, physical activity, sedentary TV viewing time, and sleep duration. Risk factors were omitted from the fully adjusted model while adjusting for all other variables. The ratio of hazard ratios (RHRs) represents the change in hazard ratios from the multivariable-adjusted model without the specific factor compared with that from the fully adjusted model. AHEI-2010, Alternative Healthy Eating Index-2010; BMI, body mass index; CI, confidence interval; SES, socioeconomic status; RHR, ratio of hazard ratio.

© 2024 Wang P et al. *JAMA Network Open*.

eFigure 5. Associations Between the Neighborhood Socioeconomic Status Score and Primary Outcomes in Never Smokers

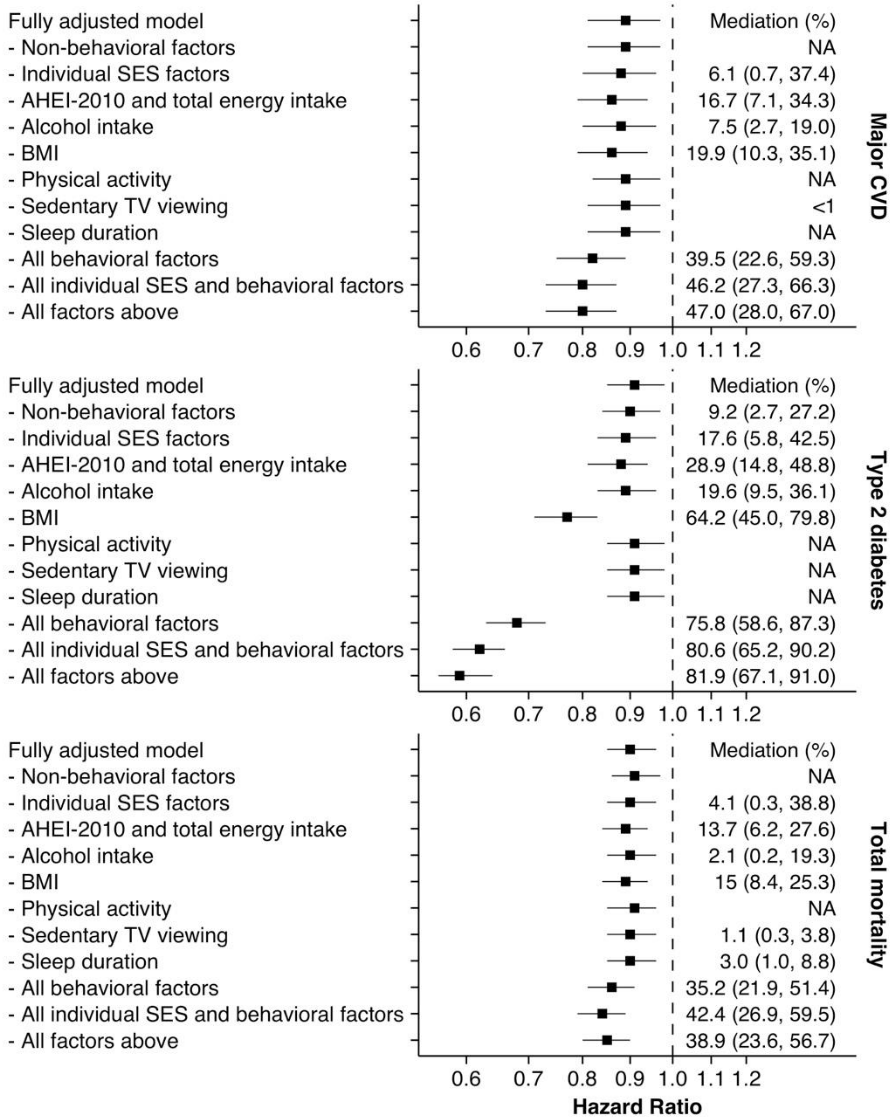

Hazard ratio and 95% confidence intervals for every 10th–90th percentile increment of neighborhood socioeconomic status score are shown on log scales. All analyses were stratified by age, calendar year, and cohort. Fully adjusted model was adjusted for dietary pattern (AHEI-2010), total energy intake, non-behavioral factors (race and ethnicity, family history of diabetes, family history of cancer, family history of cardiovascular disease, multivitamin use, regular aspirin use, regular non-steroidal anti-inflammatory drugs use, and postmenopausal hormone use for women), neighborhood SES score, individual SES factors (marital status, husband's education for women, live alone, household income for NHSII participants, working status, father's occupation for women, and mother's occupation for women), alcohol intake, BMI, physical activity, sedentary TV viewing time, and sleep duration. Risk factors were omitted from the fully adjusted model while adjusting for all other variables. Mediation proportions were not available when associations were attenuated without adjusting for hypothesized mediators compared to the fully adjusted model. AHEI-2010, Alternative Healthy Eating Index-2010; BMI, body mass index; CI, confidence interval; NA, not available; SES, socioeconomic status.

© 2024 Wang P et al. *JAMA Network Open*.
