# Supplementary material for: Socioeconomic Status, Diet, and Behavioral Factors and Cardiometabolic Diseases and Mortality
Source: JAMA Netw Open. 2024 Dec 20;7(12):e2451837. doi: 10.1001/jamanetworkopen.2024.51837 (PMC11662255; doi:10.1001/jamanetworkopen.2024.51837)
Supplement: Supplement 2. — Data Sharing Statement [file jamanetwopen-e2451837-s002.pdf]

# Data Sharing Statement

Wang. Socioeconomic Status, Diet, and Behavioral Factors and Cardiometabolic Diseases and Mortality. *JAMA Netw Open*. Published December 20, 2024.
doi:10.1001/jamanetworkopen.2024.51837

**Data**
**Data available:** No

**Additional Information**
**Explanation for why data not available:** Because of participant confidentiality and privacy concerns, data are available upon written request. According to standard controlled access procedure, applications to use the Nurses' Health Studies and Health Professionals Follow-up Study resources will be reviewed by our External Collaborators Committee for scientific aims, evaluation of the fit of the data for the proposed methodology, and verification that the proposed use meets the guidelines of the Ethics and Governance Framework and the consent that was provided by the participants. Investigators wishing to use the Nurses' Health Studies and Health Professionals Follow-up Study data are asked to submit a brief description of the proposed project. Further information including the procedures to obtain and access data from the Nurses' Health Studies and Health Professionals Follow-up Study is described at https://www.nurseshealthstudy.org/researchers (contact email: nhsaccess@channing.harvard.edu) and https://sites.sph.harvard.edu/hpfs/for-collaborators/ for details.
